# *Cyrface*: An interface from *Cytoscape* to *R *that provides a user interface to *R* packages

**DOI:** 10.12688/f1000research.2-192.v2

**Published:** 2014-07-01

**Authors:** Emanuel Gonçalves, Franz Mirlach, Julio Saez-Rodriguez

**Affiliations:** 1 The European Molecular Biology Laboratory, European Bioinformatics Institute, Cambridge, CB10 1SD, UK; 2 University of Applied Science Weihenstephan-Triesdorf, Weidenbach, 91746, Germany

## Abstract

There is an increasing number of software packages to analyse biological experimental data in the *R* environment. In particular, *Bioconductor*, a repository of curated R packages, is one of the most comprehensive resources for bioinformatics and biostatistics. The use of these packages is increasing, but it requires a basic understanding of the R language, as well as the syntax of the specific package used. The availability of user graphical interfaces for these packages would decrease the learning curve and broaden their application.

Here, we present a *Cytoscape* *app* termed *Cyrface* that allows *Cytoscape* *apps* to connect to any function and package developed in *R*. *Cyrface* can be used to run *R* packages from within the *Cytoscape* environment making use of a graphical user interface. Moreover, it can link R packages with the capabilities of *Cytoscape* and its *apps*, in particular network visualization and analysis. Cyrface’s utility has been demonstrated for two Bioconductor packages ( *CellNOptR* and *DrugVsDisease*), and here we further illustrate its usage by implementing a workflow of data analysis and visualization. Download links, installation instructions and user guides can be accessed from the *Cyrface’s* homepage ( http://www.ebi.ac.uk/saezrodriguez/cyrface/) and from the Cytoscape app store ( http://apps.cytoscape.org/apps/cyrface).

## Introduction

The availability of high-throughput experimental data has led to the development of multiple computational methods to analyse these data. One of the most used environments is the statistical programming language *R* ^1^. Multiple R packages for computational biology and bioinformatics are available in various resources such as the Comprehensive R Archive Network (CRAN). Furthermore, *Bioconductor* ^2^ provides a large collection of curated packages to analyse biological data developed in *R*. These packages are subject to stringent quality control in terms of functionality and documentation. It is an open-source project hosting 824 active and curated software packages as of May 2014.

For those not familiar with computational programming, learning *R* and using its packages can be a time consuming task. Therefore, the use of intuitive graphical interfaces that simplifies their use can enhance the usability of these R packages. *Cytoscape* ^3, 4^ is a Java open-source framework with an intuitive graphical interface devoted to the visualization and analysis of networks. It is arguably one of the most used tools in bioinformatics, and has a variety of user developed extensions to solve numerous computational biology problems. These user defined extensions are termed plug-ins (1.x and 2.x) or apps (3.x) depending of which version of Cytoscape is being used.

Here, we present *Cyrface*, an app for *Cytoscape* that facilitates an interface between any *R* package and *Cytoscape*. *Cyrface* is designed to integrate the major strengths of *R* and *Cytoscape* environments by providing a general *Java* to *R* interface. By linking these two environments, *Cyrface* allows one to use Cytoscape as a graphical user interface for *R* packages. It also enables *Cytoscape* apps to access the wealth of methods implemented in *R*.

Workflow management systems such as *Taverna* ^5^ and *Galaxy* ^6– 8^ can call R packages from a graphical user interface (GUI)-based interface. *Taverna* is a standalone *Java* open-source tool for the general development and execution of workflows. *Galaxy* is an open-source web-platform to assemble workflows based on genomic experimental data analysis. Thus, Cyrface complements Taverna and Galaxy by enhancing GUIs for *R* within a different environment with complementary features.

*RCytoscape* ^9^ is another tool that exists to link R and Cytoscape. It is a *Bioconductor R* package that establishes a connection between *R* and *Java*. The fundamental difference between RCytoscape and Cyrface is that RCytoscape supports the connection from *R* to *Java*, whereas Cyrface allows a connection from Java to *R*. A typical use of *RCytoscape* would be to handle experimental data from *R* and transfer the biological network to *Cytoscape* while controlling it within *R*. Hence, *RCytoscape* and *Cyrface* provide complementary features.

This paper is structured as follows: Firstly, we provide a description of the implementation of Cyrface. Then, to illustrate the usefulness of Cyrface, we show two existing apps, CytoCopteR ^10^ and DrugVsDisease (DvD) ^11^, that make use of Cyrface, and we demonstrate an implementation of a simplified version of the *DataRail* ^12^ workflow. Finally, we discuss on-going and future developments.

## Implementation

*Cyrface* is a Java open-source framework developed to establish the connection between *Cytoscape* and *R*. Interaction between these two different environments (invoking *R* within *Java*) is not natively supported by *Java*. Therefore, to achieve this *Cyrface* uses the external libraries *RCaller* ^13^ and *Rserve* ^14^.

On the one hand, to support the communication between *Java* and *R, RCaller* uses an *R* package called *Runiversal* that converts the *R* objects into an *XML* format, thus allowing the *R* objects to be read by *Java*.

On the other hand, *Rserve* establishes a *TCP/IP* server allowing other programs from various languages to connect to an R session and access its features. *Rserve* is currently being used by several mature projects, among them the *Taverna* workflow management system ^5^.

**Figure 1.**
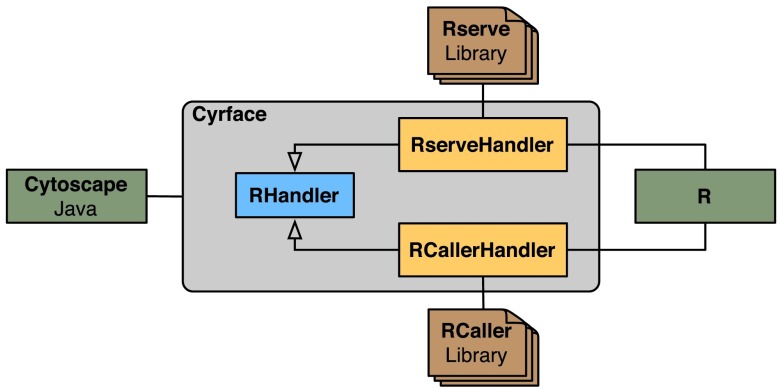
Diagram of the Cyrface interaction layer with R. Within the grey box the class hierarchy of the classes responsible for establishing the connection between Cytoscape and R is represented. RHandler is an abstract Java class that is extended by RserveHandler and RCallerHandler classes that add support to Rserve and RCaller libraries, respectively. The connection from Java to R can be achieved using either RserveHandler or RCallerHandler classes, or other classes that successfully extend RHandler.

*Rserve* and *RCaller* libraries are integrated in *Cyrface* by implementing *RserveHandler* and *RCallerHandler Java* classes, respectively. Both classes extend the abstract class *RHandler* that contains the signature of all the necessary methods to establish and maintain a connection with *R*. Figure 1 depicts the hierarchical structure of these classes and the connection points between these two different environments.

Cyrface software architecture is designed to allow the integration of other Java libraries that facilitate the connection between Java and R. Thereby, this structure allows one to take advantage of particular strengths of different libraries and to adapt to particular requirements of the users. For instance, execute R commands automatically without requiring first to manually initiate an R session.

*Cyrface* uses Cytoscape’s features, such as the Command Line. The Command Line offers the users the ability to script basic commands in Cytoscape, such as import, display or modify networks through a simple command line or script file. A useful feature of the Command Line is the ability of performing repetitive tasks automatically. By supporting this tool *Cyrface* extends the possibility of the users to integrate in their scripts methods developed in R together with common Cytoscape features. The Command Tool Dialog window can be used to dynamically execute the necessary R commands. This can be useful, for example when debugging a script.

On Cyrface’s homepage, we provide an illustrative example using the Command Line Dialog tool to plot some features of an existing and publicly available data-set termed, iris ^13^, using the well known plotting library ggplot ^14^. The iris data-set is widely used in the field of pattern recognition and machine learning and is subdivided into different classes, where each class defines the type of the plant iris. This is an illustrative example to demonstrate how Cyrface can within Cytoscape perform any task in the R environment and collect the respective output.

## Results and discussion

A typical use of *Cyrface* is to provide a graphical user interface to *R* packages within *Cytoscape*. *Cyrface* is currently being used by two *Cytoscape* plug-ins, *CytoCopteR* ^10^ and *DvD* ^11^.

*CytoCopteR* ^10^ provides a simple step-by-step interface allowing users without any experience in *R* to use the *CellNOptR* ( www.cellnopt.org) package and handle the input and output networks in *Cytoscape*. *CellNOptR* is an open-source software package that provides methods for building predictive logic models from signalling networks using experimental measurements of activation of proteins upon perturbation.

*DvD* ^11^, Drug vs Disease, is an *R* package that provides a workflow for the comparison of drug and disease gene expression profiles. It provides dynamic access to databases, such as *Array Express* ^15^, to compare drug and disease signatures to generate hypotheses of drug-repurposing.

*CytoCopteR* and *DvD* are two examples of how Cyrface captures the strengths of both environments. On one side, *R* provides a wealth of bioinformatics and biostatistics packages with very comprehensive resources such as *Bioconductor* and *CRAN*. On the other side, *Cytoscape* facilitates a user-friendly graphical interface for network visualisation and analysis, complemented with a variety of plug-ins or apps addressing different computational biological problems. *Cyrface* links these two environments by providing a way to develop user-friendly graphical interfaces for *R* packages by embedding them within *Cytoscape*.

As another illustrative example, we implemented in *Cyrface* a simplified version of the *DataRail* ^12^ workflow. This example is designed to illustrate how one can use methods already available in *R* and build a graphical user interface in Cytoscape to access them.

*DataRail* is an open-source *MATLAB* toolbox that handles experimental data in a tabular format and provides methods to maximize and extract information using internal or external tools. Experimental data is stored in a format termed Minimum Information for Data Analysis in Systems Biology ( *MIDAS*). This is a tabular format that specifies the layout of experimental data files ^12^. A typical use of *DataRail* is to import, store and process the input information from instruments using the *MIDAS* format, and export it to other *MIDAS* compliant software, such as *CellNOptR*.

**Figure 2.**
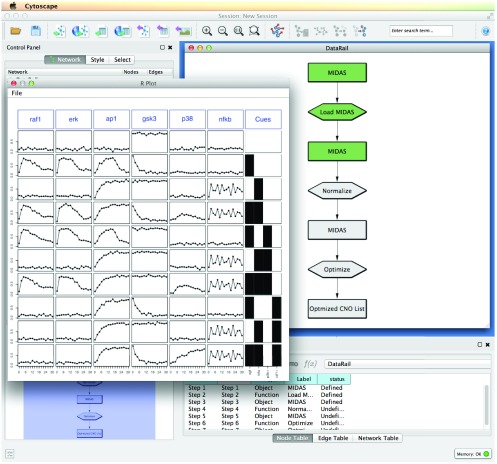
The Cyrface implementation of the DataRail ^12^ workflow. The rounded rectangles represent the MIDAS files containing the experimental data at a given state. Hexagon nodes represent functions such as load or normalise. Green identifies steps that were successfully executed and grey identifies those that were not run yet. The data-set shown represents the normalised values of the protein activity state of a set of proteins (columns) under different stimulatory conditions (rows).

The DataRail workflow implemented in Cyrface is structured in several sequential steps that allows the users to import, normalise and visualise experimental data-sets stored in the MIDAS format ( Figure 2). The workflow is tested using an *in silico* generated data-set and a signalling network from ^16^.

An extension to the workflow was subsequently added to support the model training function form *CellNOptR* package ^10^. *CellNOptR* training function maximises the fit of the experimental data and the corresponding prior-knowledge network, by generating and optimising a logic model. Thereby, through an intuitive graphical interface, users are able to select a biological network and use it to assess the quality of the fit with a corresponding data set of experimental data. This extension illustrates how one can in principle embed any R package in such a workflow, but it does not replace the *CytoCopteR* app as a complete interface for CellNOptR.

The workflow supports the SIF network format, which is supported by Cytoscape, but also the Systems Biology Markup Language Qualitative Models (SBML Qual) format ^16^. SBML Qual is an extension of the SBML format and is proposed to provide a standard representation for logic and qualitative models of biological networks. Support for importing models stored in *SBML Qual* format is achieved using the *jSBML* library ^17^ and the respective *SBML Qual* package. Supplementary material 1 provides a step-by-step tutorial on how to use the workflow.

## Conclusions

Here, we present *Cyrface*; a bioinformatics *Java* library that provides a general interaction between *Cytoscape* and *R. Cyrface* offers a way to combine a friendly graphical interface within the *Cytoscape* environment with any *R* package. A GUI should benefit beginners and occasional users; as well as being useful for training and illustration purposes, it extends the accessibility of the tool to those not familiar with the R command line interface.

Moreover, Cyrface complements other libraries such as Rserve since, (i) it is capable of using Rserve, RCaller or any other existing Java library to query R, and (ii) it provides a tailored implementation for Cytoscape, providing interfaces that are suited to Cytoscape features, such as the support of the Command Dialog tool.

*Cyrface’s* homepage (see Software Details section) contains the link to download Cyrface and user-guide instructions. A few examples demonstrating the usefulness of the tool and the different supported libraries are also shown and explained. The source-code of Cyrface is publicly available on its GitHub webpage (see Software Details section).

Future features for Cyrface will include providing connections to Cytoscape.js, improvements to the *DataRail* workflow and further developing and testing future features, such as add support access to remote servers of Rserve.

A common scenario in an interdisciplinary field such as network biology, is one where there is on one side an expert on a certain biological question, who has data to address this question and, on the other side a computational scientist who develops algorithms, but is less familiar with the experiments. To help to bridge this situation tools like Cyrface facilitate to encapsulate sophisticated algorithms developed in R in a user-friendly interface within the Cytoscape framework, to enable non-experts to apply these algorithms.

## Software details

Homepage: http://www.ebi.ac.uk/saezrodriguez/cyrface/

Software available from: http://apps.cytoscape.org/apps/cyrface

Latest source code: https://github.com/EmanuelGoncalves/cyrface

Source code as at the time of publication: https://github.com/F1000Research/cyrface

Archived source code as at the time of publication: http://www.dx.doi.org/10.5281/zenodo.10153 ^18^

License: GNU General Public License version 3.0 (GPLv3)

## Figures and Tables

**Table:** 

*Emanuel Gonçalves*
*Saez-Rodriguez Group*
*EMBL-EBI*
emanuel@ebi.ac.uk
*May 12, 2014*

## Supplementary materials

Supplementary material 1: Cyrface DataRail workflow tutorial.

## Cyrface Manual

*Cyrface is a bioinformatics Java library that provides a general interaction between Cytoscape and R. Cyrface offers a way to combine a friendly graphical interface within the Cytoscape environment with any R package. A GUI should benefit beginners and occasional users; as well as being useful for training and illustration purposes, it extends the accessibility of the tool to those not familiar with the R command line interface.*

*This tutorial is intended for Cyrface v2.0 that requires Cytoscape v3.1 and R 3.x. The following materials are all available online on Cyrface homepage:* http://www.ebi.ac.uk/saezrodriguez/cyrface

## Software Requirements

Please be sure that you have the following software installed and working:

1. R framework (version 3.x) - http://www.r-project.org/
2. Cytoscape (version 3.1.x) - http://www.cytoscape.org/

It is very important that Cytoscape version is equal or greater than 3.1.0

Linux users must run the following commands before using Cytocopter

● sudo apt-get install libcairo2-dev
  ● sudo apt-get install libxt-dev

### Introduction

http://www.ebi.ac.uk/saezrodriguez/cyrface

Cyrface ^1^ establishes an interface between R and Cytoscape ^2, 3^ by using different Java-R libraries, e.g. Rserve, RCaller. Cyrface can be used as a Cytoscape App, e.g. to run R commands within Cytoscape, or used as a library to allow your App to connect to R.

This is developed under a GNU open-source license and the source code can be accessed from the respective GitHub project.

## Installation

### Install from Cytoscape

Click Apps on Cytoscape top bar followed by Apps Manager menu then search for Cyrface and click install. This may take a few minutes.

### Install from file

Download the jar file of Cyrface from the following URL:

- www.ebi.ac.uk/saezrodriguez/cyrface

Click Apps on Cytoscape top bar followed by Apps Manager then click *Install from file*… button and search Cyrface jar file.

### Install manually

Cyrface can also be installed manually by copying the jar file mentioned before to CytoscapeConfiguration folder. CytoscapeConfiguration folder is kept in the user home folder. Drag the App jar file into the following folder:

- ~/ CytoscapeConfiguration / 3 / apps / installed /

After moving the jar file start Cytoscape.

## Cyrface DataRail Tutorial

### Introduction

This tutorial assumes that Cytoscape as well as R is already installed. The necessary files for this tutorial is a network file in SBML-Qual format and the corresponding experimental data in MIDAS format.

### Study case

To illustrate the usefulness of Cyrface we will use a simple implementation of the DataRail workflow ^4^.

The accompanying *in silico* data (MIDAS file format ^4^) replicates biologically plausible behaviour that has been seen in intracellular signalling networks, such as the transient behaviour of ERK activation and the oscillatory dynamics of NFkB translocation from the cytoplasm to the nucleus.

### MIDAS experimental data format

The MIDAS format (Minimum Information for Data Analysis in Systems Biology) ^4^ is a comma-separated file that specifies the layout of experimental data files.

Each row represents a single experimental sample; each column represents one sample attribute, such as treatment condition, or value obtained from an experimental assay.

### SBML-Qual network format

SBML-Qual format ^5^ is an extension to the System Biology Markup Language (SBML) for Qualitative Models (Qual). In one sentence, it is designed to provide a standard mean for the exchange of logical models or regulatory and signalling networks.

For more details regarding the specifications please see ^5^.

### Tutorial

1. To start Cyrface’s DataRail workflow go to *Apps* -> *Cyrface* -> *DataRail*
2. The full workflow should now be visible
3. Right-click on the top MIDAS node and then select *Cyrface* -> *Set MIDAS file*… to select the desired MIDAS file. After the MIDAS file is selected the node should turn green.
  **Raw data MIDAS file:**
  www.ebi.ac.uk/saezrodriguez/cyrface/resources/ToyDataPB10raw.csv
4. Right-click on *Load MIDAS* node and then select *Cyrface* -> *Load MIDAS*… option to load the previously selected MIDAS file. After the file is loaded the node should turn green.
5. After the MIDAS file is successfully loaded the second MIDAS node is now green showing that it’s ready to be normalized or visualized.
6. Right-click on the respective MIDAS node and the selecting the *Cyrface* -> *Plot MIDAS*… option will pop-up a plot of the data
  a. The plot SVG file can be exported click *File* -> *Save R plot*…
7. Right-click on the *Normalize* node to run the normalization function. A pop-up window will show up to allow the user to define the Normalization function arguments:
  a. **EC50Data:** parameter for the scaling of the data between 0 and 1, default=0.5
  b. **Detection:** minimum detection level of the instrument, everything smaller will be treated as noise (NA), default to 0
  c. **Saturation:** saturation level of the instrument, everything over this will be treated as NA, default to Inf
8. After normalizing the MIDAS file it can be plotted as previously and/or exported.

### Optional steps

Cyrface’s DataRail ^4^ Workflow is also linked to the CellNOptR ^6^ R package allowing the users to optimize a selected prior knowledge network against the just normalized MIDAS file.

9. Right-click on the *Optimize* node and select *Cyrface* -> *Optimize*… function will pop-up a file browser to select the model file. Both SIF and SBML-Qual ^5^ formats are supported.
10. The optimization may take awhile and it’s executed using the defaults values defined in CellNOptR
11. Right-click on the *Optimized CNO List* will show how well the optimized model fit the data.
12. For more details about the normalization function and the optimization method please visit CellNOptR package in Bioconductor or CellNOpt homepage
  a. www.bioconductor.org/packages/2.12/bioc/html/CellNOptR.html
  b. www.cellnopt.org

## Links

- *Cyrface* - http://www.ebi.ac.uk/saezrodriguez/cyrface
- *Cyrface (Github)* - https://github.com/EmanuelGoncalves/cyrface
- *Cytoscape* - http://www.cytoscape.org/
- *R* - http://www.r-project.org/
- *Rserve* - http://www.rforge.net/Rserve/
- *Rcaller* - http://www.mhsatman.com/rcaller.php
