# Supplementary material for: Cyrface: An interface from Cytoscape to R that provides a user interface to R packages
Source: F1000Res. 2014 Jul 1;2:192. Originally published 2013 Sep 19. [Version 2] doi: 10.12688/f1000research.2-192.v2 (PMC3962008; doi:10.12688/f1000research.2-192.v2)
Supplement: Peer review history

# Peer review: Referee response for version 2

**DOI:** 10.5256/f1000research.4521.r5322

**Published:** 2015-08-17

**Authors:** Ghislain Bidaut

**Affiliations:** 1 Integrative Bioinformatics, Inserm, Centre de Recherche en Cancérologie de Marseille, Marseille, France

My main concerns from the first version were taken into account and the paper subsequently corrected.

I have no further comments.

I have read this submission. I believe that I have an appropriate level of expertise to confirm that it is of an acceptable scientific standard.

# Peer review: Referee response for version 1

**DOI:** 10.5256/f1000research.2371.r1863

**Published:** 2014-01-20

**Authors:** Paul Shannon

**Affiliations:** 1 Computational Biology, Fred Hutchinson Cancer Research Institute, Seattle, WA, USA

Cyrface is a welcome addition to the Cytoscape ecosystem; nicely complementary to RCytoscape. My only reservation is one which applies to my own work (the aforementioned RCytoscape), indeed as well as all parts of the Cytoscape ecosystem.

My reservation has two parts:

First: network biology is in its infancy and as such experimental data are woefully incomplete. Molecular interactions are stochastic, contingent and often very short-lived yet it is exactly these molecular interactions that we need to understand in order to predict and control cellular activity in health and disease.

Second: it is (and may remain) unusual to find researchers, much less clinicians, who are adept at both programming and biomedicine. These two disciplines seem to select for, and then reinforce, different styles of thinking. Therefore progress in this field (call it network biology, or systems biology, or integrative biomedicine) requires hybrid teams: some who are very strong in biological and/or clinical sensibilities and some who are strong in computation and data analysis. Such a hybrid team, at its best, stays together long enough for mutual understanding and communication to emerge, as in the "trading language" which emerged in the world of particle physics in and around linear accelerators in the 60's (see Peter Galison's "Image and Logic").

I worry about the following scenario for Cyrface: a capable programmer hooks up the latest and greatest Bioconductor package to Cytoscape, exposes as best they can the parameterizations offered by that package, and turns the tool over to their collaborating biologist. Experimental data is loaded and analyses or simulations run. Puzzles and inconclusive results will inevitably emerge, requiring detailed knowledge of both the strengths and weaknesses of the Bioc package. With good luck, perseverance and good data, this small working team may in time settle on a satisfactory Cyrface tool which can be reused without the constant intervention of the programmer. This will last until new data is acquired, upsetting the equilibrium, and the hybrid style of work and the back-and-forth between biologist and programmer, begins anew.

I say that I worry about this scenario. It may be exactly the intended use of Cyrface; the problem it is intended to solve. But this essentially sociological characteristic (requirement?) of Cyrface is not described in the paper. I think that those of us who create bioinformatics software tend to avoid being explicit about this - and I think that this (the social & collaborative requirements of bioinformatic research) deserves a lot more attention. 

If indeed network biology, as I claim, is in its infancy, then it may be helpful if the ecosystem of Cytoscape-related tools are considered from this perspective. I suspect that the conclusions we might all (mostly) agree upon are:

1. User-friendly exploration of data-rich networks in a web browser (as with cytoscape.js) will become increasingly popular.
2. That user-friendliness often competes with analytical nuance and close scrutiny - biologically and clinically useful results become less likely.
3. Cyrface's connection of Cytoscape to R is a great step in the right direction, marrying as it does user-friendliness with some new analytical power in a way that is nicely complementary to Cytoscape java plugins and Cytoscape access to web services.

Thus, Cyrface is a good step in the right direction. The next steps, it seems to me, include:

1. Providing easy connections to R (python, C++) analyses for cytoscape.js
2. A standard mechanism whereby scripts (R, python, Ruby, Perl)  upon execution, can start up a Cytoscape or cytoscape.js session, customize it with networks, functions, buttons and menus, and with both public and laboratory data. As a generalization of Cyrface, this mechanism would encourage the rapid expansion of Cytoscape capabilities.

These possible next steps carry on in the spirit of Cyrface, RCytoscape, and Cytoscape3 apps, and will promote the creation of, and sharing of, custom network analyses, shared tools, and lead to fruitful collaborations across the hybrid community of biologists, physicians and programmers.

I have read this submission. I believe that I have an appropriate level of expertise to confirm that it is of an acceptable scientific standard.

# Peer review: Referee response for version 1

**DOI:** 10.5256/f1000research.2371.r2157

**Published:** 2013-12-12

**Authors:** Ghislain Bidaut

**Affiliations:** 1 Integrative Bioinformatics, Inserm, Centre de Recherche en Cancérologie de Marseille, Marseille, France

The paper is well written, however, many items require clarification:

1. My main criticism is the initial need to install several packages (RCaller, Rserve, Runiversal, Java, etc...), plus Cytoscape and the CommandTool plugin. Since I did not know if this would break my personal configuration or if I would be able to uninstall it, I was not able to performe the whole installation myself. It would be great if the authors could provide a virtual machine image with all the software preloaded so that one can try it out of the box without installation. I know by experience that cityscape plugins tend to work only with a single version of Cytoscape, so a clear list of all the required versions in the paper itself would be very useful.
2. The number of packages we are dealing with is very confusing. I am also afraid that with such a large number of dependencies, the program may break after any update.
3. In the Implementation section, the authors mention the "iris dataset". It would be useful to define what this is. It is also mentioned in the documentation but it is still unclear what the authors are referring to. The documentation shows an example where the ggplot2 package is used to plot "petal". Could the authors please define what this is?
4. Figure 2: It would be useful to define more accurately what type of data we are looking at here (e.g. gene expression?).
5. In the discussion, several R packages are mentioned (DvD, Cytocopter), what are their links with the present software other than the fact that they run under R? Is it really possible to interact with them from Cytoscape? If so, the proper documentation or a tutorial should be provided. It would also be useful if an actual example using DvD along interaction data, could be shown.
6. I do not understand the example with DataRail. The usefulness of the example given is not clear to me since it seems that no interaction data is given (for me this is the main purpose of Cytoscape). A useful example for most users might be:
  - A network of protein-protein interaction (PPI) data in Cytoscape.
  - Some expression data in R (an exprs object for instance).
  An example of a question might be " *How to superimpose the expression data and generate a proper network attribute from it?*"

I have read this submission. I believe that I have an appropriate level of expertise to confirm that it is of an acceptable scientific standard, however I have significant reservations, as outlined above.

# Peer review: Referee response for version 1

**DOI:** 10.5256/f1000research.2371.r1866

**Published:** 2013-10-09

**Authors:** Nikolaus Schultz, B. Arman Aksoy

**Affiliations:** 1 Computational Biology Center, Memorial Sloan-Kettering Cancer Center, New York, NY, USA; 2 Memorial Sloan-Kettering Cancer Center, New York, NY, USA

In this manuscript, the authors describe the Cytoscape plugin Cyrface. Cyrface consists of two components: 1) a Java API, which is already being used by the Cytoscape plugins CytoCopteR and DrugvsDisease (both developed by the same group), and 2) a graphical user interface that connects R to Cytoscape. As a proof-of-concept to what kind of applications can be built on top this interface, the plugin also supports the MIDAS and SBML-Qual formats.

The article is well written and the tool is useful to the community. However, we recommend the following changes to the article to make it more appealing to potential future users:

 

1. As the new version of the Cytoscape (3.x) is becoming more widely used by the community, the authors should explicitly state that they are targeting version 2.8 with this framework. This will reduce the confusion for users who are not as familiar with Cytoscape.
2. The tutorial in the supplementary materials helps to understand the general use case for this plug-in, but the lack of downloadable “sample” files for this example will make it harder for users to learn how to use the DataRail pipeline. We think it is important to provide example files that people can use for reproducing the figures in the manuscript.
3. ​The Cyrface interaction layer with R looks helpful for programmers, but can the authors comment on how these classes are different from the default Java implementations of the RServe clients, e.g. http://rforge.net/RServe? This will help clarify why people should use Cyrface for their next project.
4. The command line interface (commandTool) appears to be useful; but it seems that it is only capable of running commands in an isolated environment, with each command having its own session. If this is the case, can the authors comment on what the advantage of running R commands from the commandTool is compared to initiating a terminal window and running commands directly from an R shell? Are users able to, for example, pass node/edge attribute fields to the corresponding R commands?
5. It looks like the current implementation does not support setting a different RServe location other than localhost. Although not necessary, if users are given the option to set a different RServe address within the plug-in, this will further lower the barrier for users who are not experienced with R to use Cyrface, where they can use a pre-installed Rserve hosted on a different machine.

We have read this submission. We believe that we have an appropriate level of expertise to confirm that it is of an acceptable scientific standard.
